# Proper migration and axon outgrowth of zebrafish cranial motoneuron subpopulations require the cell adhesion molecule MDGA2A

**DOI:** 10.1242/bio.20148482

**Published:** 2015-01-08

**Authors:** Esther Ingold, Colette M. vom Berg-Maurer, Christoph J. Burckhardt, André Lehnherr, Philip Rieder, Philip J. Keller, Ernst H. Stelzer, Urs F. Greber, Stephan C. F. Neuhauss, Matthias Gesemann

**Affiliations:** 1 Brain Research Institute of the University Zurich and Swiss Federal Institute of Technology (ETH), Department of Biology, 8057 Zurich, Switzerland; 2 Institute of Molecular Life Sciences, University of Zurich, 8057 Zurich, Switzerland; 3 EMBL Heidelberg, Meyerhofstraße 1, 69117 Heidelberg, Germany

**Keywords:** Zebrafish, MDGA, Cranial motoneurons, Digital light sheet microscopy, Cell migration, Cell adhesion, Axon guidance

## Abstract

The formation of functional neuronal circuits relies on accurate migration and proper axonal outgrowth of neuronal precursors. On the route to their targets migrating cells and growing axons depend on both, directional information from neurotropic cues and adhesive interactions mediated via extracellular matrix molecules or neighbouring cells. The inactivation of guidance cues or the interference with cell adhesion can cause severe defects in neuronal migration and axon guidance. In this study we have analyzed the function of the MAM domain containing glycosylphosphatidylinositol anchor 2A (MDGA2A) protein in zebrafish cranial motoneuron development. MDGA2A is prominently expressed in distinct clusters of cranial motoneurons, especially in the ones of the trigeminal and facial nerves. Analyses of MDGA2A knockdown embryos by light sheet and confocal microscopy revealed impaired migration and aberrant axonal outgrowth of these neurons; suggesting that adhesive interactions mediated by MDGA2A are required for the proper arrangement and outgrowth of cranial motoneuron subtypes.

## INTRODUCTION

During the formation of the nervous system subsets of postmitotic neuroblasts delaminate from primary neuroepithelia and migrate over considerable distances to settle in different regions of the growing organism. These migratory cells follow stereotypic pathways detecting even miniature concentration differences of attractive and repulsive neurotropic cues or changes in the adhesive properties of the surrounding tissue. Therefore miss-regulation of either neurotropic or adhesive molecules often leads to aberrant migration and ectopic clustering of neuronal populations.

Following neuronal migration and terminal differentiation, neurons have to properly connect with corresponding targets in order to integrate the information flow throughout the nervous system. In analogy to neuronal migration, elongating neurites also grow along predetermined pathways relying on instructional information from attractive and repulsive cues and growth promoting adhesive environments. Cell-cell and cell-substrate interactions via adhesion molecules, especially by molecules belonging to the immunoglobulin superfamily, have been shown to be crucial for nervous system development (Maness and Schachner, 2007). This has been demonstrated by countless *in vitro* assays, antibody perturbation assays as well as loss and gain of function experiments (Bingham et al., 2002).

Due to its relatively simple segmental organization, the developing hindbrain has been the focus of many studies (Bingham et al., 2010). As hindbrain development is essentially conserved among vertebrates, knowledge derived from one species can potentially give insights into hindbrain development in other species (Gilland and Baker, 1993; Moens et al., 1998; Moens and Prince, 2002; Gilland and Baker, 2005). In our study we have focused on the development of the zebrafish hindbrain, especially studying migration and axonal outgrowth of branchiomotoneurons (Drapeau et al., 2002). The concise and segmental organization as well as the stereotype migration and axonal outgrowth pattern have made branchiomotoneurons an attractive model system. Branchio- as well as somato- and viscera-motoneurons represent subgroups of cranial motoneurons whose axons exit the CNS at predetermined exit points (for reviews, see Chandrasekhar, 2004; Song, 2007). Neurons from specific nuclei form different cranial nerve bundles innervating the muscle masses of the branchial (pharyngeal) arches. While somatomotoneurons, innervating extraocular muscles, cluster in the oculomotor (cranial nerve III), the trochlear (IV) and the abducens (VI) motor nuclei; branchiomotoneurons (BMN) build up the trigeminal (V), facial (VII) glossopharyngeal (IX) and vagal (X) nuclei. In zebrafish BMN migration and axon outgrowth is initiated within the first 24 h of development. BMN precursors are generated in specific rhombomeres, which subsequently migrate towards their final destination at characteristic dorsolateral and rostrocaudal positions within the developing hindbrain. For example motoneuron precursors of the facial nerve originating in rhombomere 4 migrate as far as rhombomeres 6 and 7 (Chandrasekhar, 2004; Song, 2007). These cells project axons via specific motor nerves into the periphery. The generation of transgenic zebrafish where GFP expression is driven by the islet1 promoter has proven valuable to study the generation, positioning and axon outgrowth of branchiomotoneurons (Higashijima et al., 2000).

Using this Isl1-GFP transgenic line the involvement of planar cell polarity (PCP) pathway genes such as Stbm/Vangl2/tri (Jessen et al., 2002; Sittaramane et al., 2009), prickle1a (Carreira-Barbosa et al., 2003), prickle1b (Rohrschneider et al., 2007), scribble1 (Wada et al., 2005), Celsr2 and Frizzled3a (Wada et al., 2006), col/hdac1 (Nambiar et al., 2007), as well as the PCP effector gene Nhsl1b (Walsh et al., 2011) in the migration of branchiomotoneurons has already been demonstrated. However, besides the genes from the planar cell polarity pathway, other factors must be involved in motoneuron migration in the hindbrain, as several aspects of the migration appear normal in vangl2 mutants (Bingham et al., 2010). Furthermore, a “collective mode” of migration that requires the interaction between migrating facial BMNs themselves and is independent of PCP proteins has been suggested to work together with PCP-dependent mechanisms to drive directed migration of facial BMNs *in vivo* (Walsh et al., 2011). Recent studies also suggest that fucosylated glycans, such as gmds/twd expressed by neuroepithelial cells (Ohata et al., 2009), may repulse migrating vagal motoneurons preventing radial/apical migration (Ohata et al., 2011). Moreover, TAG1, laminin and cadherin mediated signals have been shown to be involved in guiding branchiomotoneurons (Sittaramane et al., 2009; Grant and Moens, 2010; Stockinger et al., 2011). In addition, interaction between motor nerves and sensory nerves are required for the proper axonal growth of trigeminal but not facial nerves (Cox et al., 2011), but the molecules mediating this interaction remain unknown. Interestingly, recently it has been shown that facial branchiomotoneuron migration also depends on the interaction of migrating neurons with axons of the medial longitudinal fascicle (MLF), as preventing MLF axons from entering the hindbrain results in staling of FBMN migration (Wanner and Prince 2013).

We have recently identified a novel group of cell adhesion molecules, called MDGAs (for MAM domain containing glycosylphosphatidylinositol anchor proteins) (Gesemann et al., 2001; Litwack et al., 2004). MDGAs, which belong to the immunoglobulin superfamily of cell adhesion molecules (for review, see Maness and Schachner, 2007), have been shown to be expressed in the spinal cord of different species including rat (Litwack et al., 2004), chicken (Joset et al., 2011) and medaka (Sano et al., 2009). For chicken it has been demonstrated that inactivation of MDGA2 by RNA interference or function blocking antibodies leads to outgrowth defects of commissural interneurons (Joset et al., 2011). Subsequent experiments have demonstrated that MDGA2 interacts homophilically and that interactions between commissural interneurons and MDGA2 positive ipsilateral projecting neurons are important for proper rostral growth of commissural interneurons (Joset et al., 2011).

In order to explore additional roles of MDGA in nervous system development, we have now analyzed MDGAs in the zebrafish *Danio rerio*. In zebrafish three different MDGAs are present; MDGA1, MDGA2A and MDGA2B. Of these, MDGA2A is highly expressed in subsets of migrating cranial motoneurons. Using morpholino mediated knockdown experiments, we could demonstrate that the absence of MDGA2A leads to migration defects of trigeminal neurons as well as aberrant axonal growth and defasciculation of facial branchiomotor axons.

## RESULTS

Through comparative database searches with rat, human and chicken MDGA sequences, we found and subsequently cloned three orthologous zebrafish MDGA genes, namely MDGA1, MDGA2A and MDGA2B (for a phylogenetic comparison, see supplementary material Fig. S1). The amino acid sequence of zebrafish MDGA1 shows 59% and the one of zebrafish MDGA2A and -2B 76% and 74% identity with the corresponding rat orthologs. As the homology between individual domains of MDGAs varies significantly, a detailed homology analysis is given in supplementary material Fig. S1. While some data about the RNA distribution and functional properties of rat (Litwack et al., 2004; Takeuchi and O'Leary, 2006; Takeuchi et al., 2007), mice (Ishikawa et al., 2011), chicken (Fujimura et al., 2006; Joset et al., 2011) and medaka (Sano et al., 2009) MDGAs is available, no such information exists for the zebrafish embryo. We therefore performed in situ hybridization assays in zebrafish embryos at different stages of development, to test whether MDGA gene expression correlates with specific aspects of nervous system development.

### Zebrafish MDGAs are expressed in the spinal cord and in defined brain areas

**Fig. 1.**
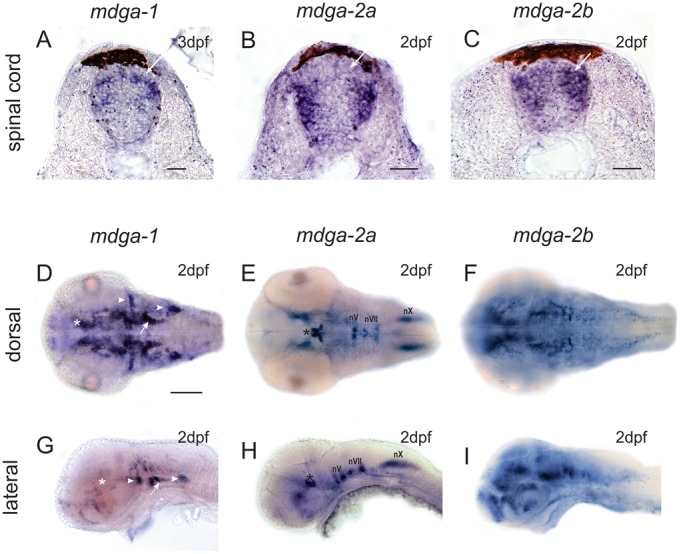
MDGAs are expressed in the developing zebrafish spinal cord and brain. (A-C) Cross-section through larval zebrafish spinal cords. MDGA1 and MDGA2B staining can be observed at regions where dorsal commissural interneurons are located (white arrow). Additional staining for MDGA2A and MDGA2B can be observed in pools of intermediate and ventral interneuron. MDGA1 positive cells can be found in a narrow band of mediolateral located interneurons. Scale bars represent 25 µm. (D–F) Dorsal and (G–I) lateral view of MDGA whole mount in situ hybridizations of 2dpf larval zebrafish. MDGA1 riboprobes label the ventral thalamus (asterisks in D,G) and the hypothalamus (arrow in D,G). Three well discernible cell clusters in the peripheral nervous system, namely the anterior and posterior lateral line ganglia as well as cells associated with the otic placode such as cells of the statoacoustic ganglion (gVIII) also prominently express MDGA1 (arrowheads in D,G). MDGA2A transcripts can be found in the motoneurons of the oculomotor (nIII) and trochlear (nIV) nerve in the midbrain (asterisks in E,H), as well as branchiomotoneurons of the trigeminal, facial and vagal nerve in the hindbrain (E,H, nV/nVII/nX). MDGA2B is expressed in the telencephalon, the ventral thalamus, the tegmentum, the hypothalamus and at low levels also in subpopulation (nVII and nX) of cranial motoneurons (F,I). Scale bars in D equals 100 µm.

Since rat and chicken MDGAs are highly expressed in the developing spinal cord (Litwack et al., 2004; Joset et al., 2011), we started our analysis in the corresponding region of the zebrafish embryo. As expected from rat and chicken studies, zebrafish MDGA transcripts are expressed in distinct interneuron subpopulations within the dorsal and mediolateral part of the embryonic spinal cord (Fig. 1A–C). MDGA1 and MDGA2B transcripts can be observed at regions where dorsal commissural interneurons are located. In addition, MDGA2A and MDGA2B messages are expressed in cell pools that coincide with the location of intermediate and ventral interneuron subpopulations. In the spinal cord MDGA1 positive cells can further be found in a narrow band of mediolateral located interneurons as well as within the dorsal ventricular zone (Fig. 1A), from where newborn cells spread laterally.

Interestingly, MDGA transcripts are also abundantly expressed in the zebrafish brain. At two days post fertilization, MDGA1 riboprobes label distinct clusters of neurons that stretch bilaterally alongside the medial border of the eyes (Fig. 1D,G). The anterior region encompasses the ventral thalamus (asterisks in Fig. 1D,G) and the hypothalamus (arrow in Fig. 1D,G), whereas the posterior cell clusters represent low level staining of branchiomotoneurons. MDGA1 transcripts are also highly expressed in cells of the peripheral nervous system. Three well discernible cell clusters, the anterior and posterior lateral line ganglia as well as cells associated with the otic placode such as cells of the statoacoustic ganglion (gVIII) prominently express MDGA1 (arrowheads in Fig. 1D,G).

MDGA2A transcripts are localized in distinct neuronal clusters corresponding to motoneurons of several cranial nerves (Fig. 1E,H). Among them are motoneurons of the oculomotor (nIII) and trochlear (nIV) nerve in the midbrain (asterisks in Fig. 1E,H), as well as BMNs of the trigeminal, facial and vagal nerve in the hindbrain (Fig. 1E,H, nV/nVII/nX). In addition, MDGA2A transcripts are also weakly expressed in muscle tissue, such as the sternohyoideus (sh) and in mandibular muscles (data not shown). Expression in head muscles can also be seen for MDGA2B transcript. In addition, MDGA2B is expressed in the telencephalon, the ventral thalamus, the tegmentum, the hypothalamus and at low levels also in subpopulation of cranial motoneurons (Fig. 1F,I).

### MDGA2A is expressed in axonal tracts of cranial motoneurons during development

**Fig. 2.**
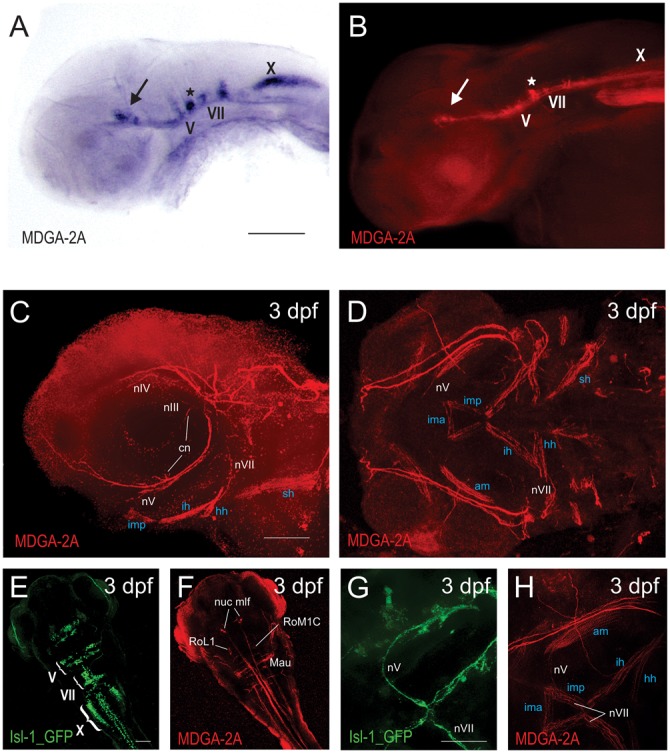
Antibodies against MDGA2A label neurons and axons of branchiomotoneurons. (A,B) Comparison between MDGA2A RNA and protein distribution demonstrates that the MDGA2A antibody highlights identical structures as seen with MDGA2A riboprobes. Neurons and axons of the oculomotor (arrow), the trigeminal (V) and the facial (VII) nerve are clearly MDGA2A positive (A,B). (C,D,F,H) At 3dpf MDGA2A is located on specific nerves. MDGA2A antibodies highlight branches of the ciliary nerve (cn), the oculomotor nerve (nIII), the trochlear cranial nerve (nIV), several branches of the trigeminal nerve (nV) and the facial nerve (nVII) (C,D). MDGA2A positive nV axons run ventrally along the caudal edge of the eye. The distal nV innervates the imp and merges with both the contralateral nV and the distal tip of the seventh nerve (nVII) at the midline (H). Moreover neurons and axons of medial longitudinal fascicle (mlf), the Mauthner neurons (Mau) and neurons of the reticular formation (RoM1C, RoL1) express MDGA2A (F). (E,G) Isl-1 GFP staining in cranial motoneurons. As previously reported Isl-1 GFP transgenic zebrafish display intense cranial motoneurons V, VII and X staining (E). Moreover staining in the trigeminal as well as the facial nerve can easily be seen (G). Abbreviations: am, adductor hyomandibulae; cn, ciliary nerve; hh, hyohyal muscle; ih, interhyal muscle; ima, intermandibularis anterior; imp, intermandibularis posterior; Mau, mauthner neurons; nuc, mlf nucleus of the medial longitudinal fascicle; nV, trigeminal nerve (neurons); nVII, facial nerve (neurons); nX, vagal nerve (neurons); RoL1 and RoM1C, neurons of the reticular formation; sh, sternoid muscle. Scale bars equal 100 µm.

The concise and segmentally arranged cell bodies and their stereotype axonal course have made BMNs an easily accessible system for studying different aspects of neuronal development, such as tangential migration and axon pathfinding (Bingham et al., 2002). Moreover, transgenic zebrafish lines with BMNs expressing GFP under the control of the Islet 1 promoter have substantially contributed to deciphering the molecular aspects of their development. In this transgenic zebrafish line, the Islet1 promoter/enhancer sequence drives GFP expression in cranial motoneurons, some of the cranial sensory neurons, and several other groups of cells (Fig. 2E,G; Higashijima et al., 2000). As MDGA2A is highly expressed in cranial motoneurons, we generated peptide antibodies against MDGA2A to analyze the distribution and potential role of MDGA2A in developing cranial neurons. In agreement with the RNA distribution pattern, MDGA2A antibodies stained neuronal cell bodies and axonal tracts and to a lesser extend head muscles. In the hindbrain, branchiomotoneurons are stained by MDGA2A antibodies (Fig. 2A,B). Among the MDGA2A positive cranial nerves are tracts of the oculomotor (nIII) and trochlear (nIV) nerves. The ciliary nerve (cn, part of nIII) innervating the lens muscle as well as a branch of nIV that innervates the superior oblique muscle are clearly MDGA2A positive (Fig. 2C). Additional axons expressing MDGA2A are the trigeminal (nV) and facial (nVII) motor nerves, innervating muscles in the jaw and jaw-support structures (Schilling and Kimmel, 1997; Chandrasekhar, 2004), as well as axons within the vagal motor nerve (nX) (Fig. 2C,D,H).

In addition to structures in the hindbrain, the MDGA2A antibody recognizes a number of neurons and axons in the central and peripheral nervous system (Fig. 2F; supplementary material Fig. S2). Among them are axons of the presumptive spino-occipital nerve (supplementary material Fig. S2), the bilaterally arranged nuclei of the medial longitudinal fascicle (nuc mlf; Fig. 2F) and its axonal tracts (mlf; supplementary material Fig. S2), reticulospinal neurons such as the assumed RoM1C and RoL1 neurons as well as the large Mauthner neurons. Moreover, the lateral line system and components of the statoacoustic ganglion, both of which innervate hair cells of the head, trunk, tail and the inner ear are MDGA2A positive (for more information, see supplementary material Fig. S2).

In summary, MDGA2A protein is prominently expressed in several neuronal subpopulations and their axons during the period of neuronal migration and axon outgrowth, suggesting a potential role for this molecule in mediating adhesive interactions during these processes. To test such a hypothesis, we selectively downregulated MDGA2A protein synthesis using morpholino antisense oligonucleotides and analyzed occurring phenotypes.

### MDGA2A deprivation leads to impaired migration and axonal growth of branchiomotoneurons

**Fig. 3.**
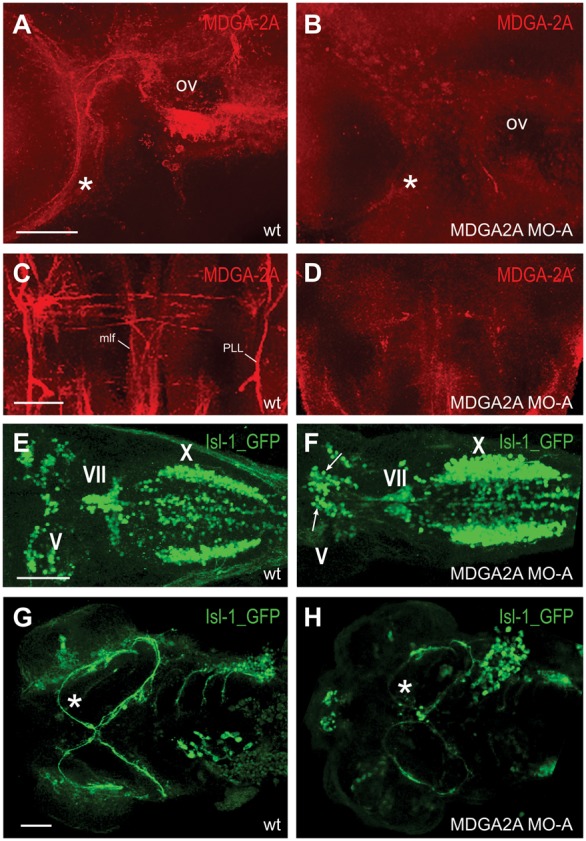
MDGA2A knockdown causes migration and guidance errors of trigeminal motoneurons. (A–D) MDGA2A staining in wt and MDGA2A knockdown zebrafish at 3dpf. Lateral views of MDGA2A labeled zebrafish demonstrate that antisense morpholino injections against MDGA2A efficiently downregulates the expression of MDGA2A. Neither MDGA2A positive hair cells along the otic vesicle (oc) nor the trigeminal nerve (asterisk) are stained in MDGA2A morphants (A,B). Also staining in the mlf and PLL is clearly downregulated (C,D). (E–H) Migration and axon outgrowth pattern of branchiomotoneurons in wt and MDGA2A knockdown zebrafish at 3dpf. In wt fish branchiomotoneurons occupy characteristic locations, with the trigeminal cluster being located in r2/r3 and the facial cluster lying adjacent to the midline (E). In MDGA2A morphants, trigeminal neurons settle at ectopic locations (arrow) (F). Moreover, staining intensity in the trigeminal (asterisk) and facial (arrowhead) nerve is strongly reduced in MDGA2A knockdown larva, suggesting that fewer axons are present in these nerve bundles. Abbreviations: ov otic vesicle; mlf medial longitudinal fascicle; PLL posterior lateral line fascicle; V trigeminal motoneurons; VII facial motoneurons; X vagal motoneurons. Scale bars in A,B, and E–H equal 50 µm, in C,D 25 µm.

Since MDGA2A message is present in subgroups of branchiomotoneurons, namely the trigeminal, facial and vagal motoneurons, we further analyzed the effect of MDGA2A protein knockdown in Islet1-GFP transgenic zebrafish embryos. To analyze the efficiency of protein knockdown in MDGA2A morpholino treated zebrafish we compared antibody stainings in wildtype (wt) and MDGA2A knockdown animals. Neurons and axons that are clearly stained in wt fish, lack corresponding immunoreactivity in MDGA2A knockdown zebrafish (Fig. 3A–D; supplementary material Fig. S4C). Interestingly, several cranial motoneuron migration and axon outgrowth defects could be observed in Islet1-GFP MDGA2A knockdown animals. While in wt zebrafish migration of trigeminal BMNs occurs within the limits of their rhombomere (r) of origin resulting in the formation of an anterior and posterior trigeminal motor nucleus in r2 and r3, respectively; in MDGA2A morphants, trigeminal motoneurons (V) fail to undergo their proper migration pattern and settle at various ectopic positions (arrows in Fig. 3F). Also, neurons within clusters appear disorganized, accumulating in a pile, instead of settling in the characteristic lateral trigeminal clusters. Interestingly, motoneurons of the facial nerve (VII) have left their place of origin in r4 and undergone appropriate caudal migration. However, their number in the forming clusters seems to be reduced. Moreover, the bilateral branches of the vagal motor nucleus appear more prominent, as if containing more cells than normal.

Examination of the corresponding axon tracts in uninjected and morphant embryos suggest that fewer axons run in axonal tracts of MDGA2A deprived embryos (Fig. 3G,H). Interestingly, their course and innervation pattern is still maintained in MDGA2A knockdown animals, indicating that proper pathfinding of growing trigeminal and facial motoneurons can still occur. Nevertheless, many axons seem to stall along the axonal path, leaving a reduced number to reach their muscle targets. While the reduced number of axons running in these tracts may be caused by errors originating from the absence of MDGA2A, this phenotype may also be caused by migration defects of the mentioned branchiomotoneurons. In order to study these phenotypes in more detail *in vivo*, we performed time- lapse light sheet microscopy.

### Live light sheet microscopy of zebrafish branchiomotoneuron development confirms MDGA2A knockdown phenotypes

**Fig. 4.**
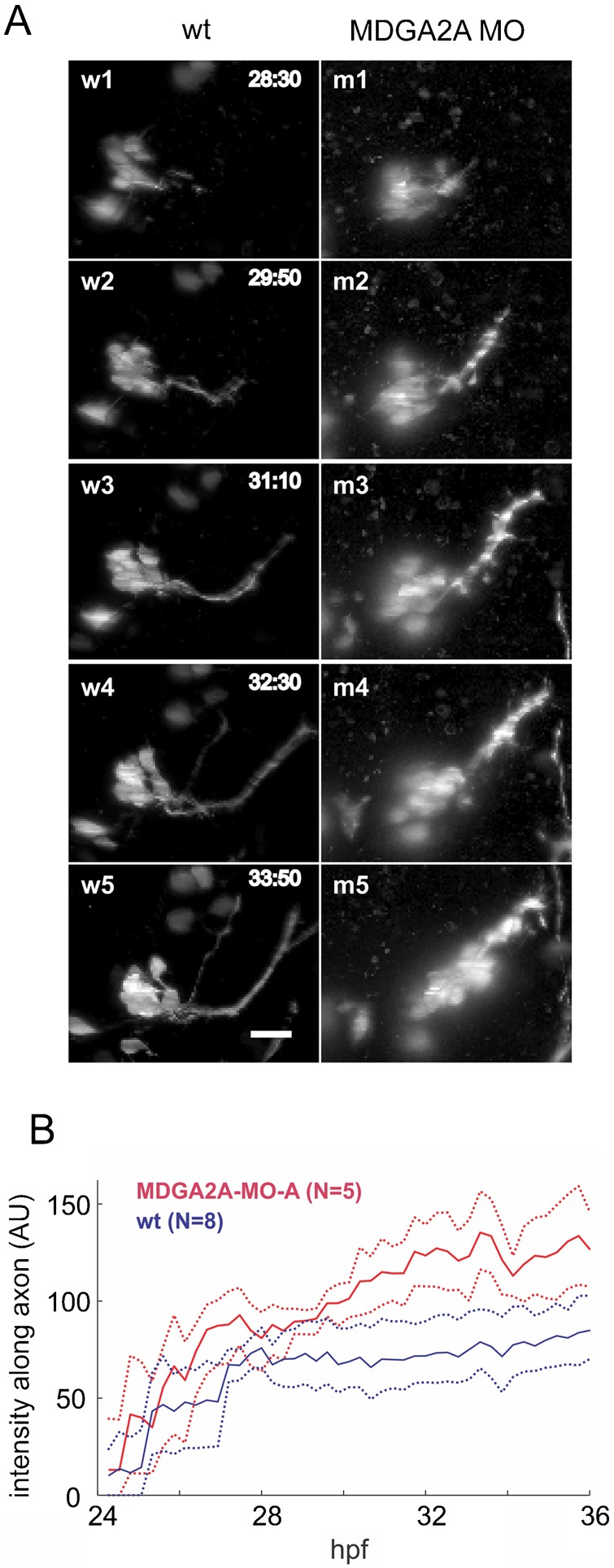
MDGA2A knockdown increases mobility of trigeminal (V) neurons. (A) Living isl-1 embryos were imaged by digital scanned laser light sheet microscopy from 24–36 hpf at 16 min intervals. For analysis the images were deconvolved and the stacks were compressed into maximum projections. Representative images from control and MDGA2A morpholino treated embryos are shown. During the experimental period trigeminal neurons send out axons along a well-defined path, with no movement of the trigeminal neurons (w1–w5). In MDGA2A knockdown animals the compactness of the trigeminal cell cluster is impaired. Trigeminal neurons migrate along their axon bundles leaving their place of origin (bright dots along the axon bundle; m1–m5). (B) To quantify this migration phenotype, the time course of the fluorescence intensity along the trigeminal axon bundle was quantified in 8 control embryos and 5 MDGA2A morpholino treated embryos. Shown are the mean and bootstrap confidence intervals (as dotted lines, α = 0.05). Note that the fluorescence intensity along the trigeminal nerve in MDGA2A knockdown animals increases significantly past 30 hpf, representing the aberrant migration of trigeminal neurons. For more details see supplementary material Fig. S3A–C for individual intensity traces. The timestamp is hours post fertilization (h: min), N is the number of analyzed embryos, the scale bar is 20 µm.

To observe branchiomotoneuron migration and axon outgrowth in real time, Isl-l:GFP fish were imaged by digital scanned light sheet microscopy (DSLM) between 24–36 hpf. Image stacks that covered the region of the nV, nVI and nVII axons were recorded every 16 min (supplementary material Movie 1). In wt control embryos trigeminal neurons formed compact clusters and stayed together throughout the period of observation (24–36 hpf; Fig. 4A, w1–w5; supplementary material Movie 2). Interestingly, in MDGA2A morpholino treated embryos, single trigeminal neurons started to move along the axon bundle instead of remaining in a stable cell cluster (Fig. 4A, m1–m5; supplementary material Movie 2). To quantify this phenotype we measured the fluorescence intensity along the trigeminal nerve. At 28 hpf the fluorescence intensity along the axons started to increase in MDGA2A knockdown animals compared to control embryos and remained higher than control values during the rest of the observation period. During this period continuous ectopic migration takes place in morpholino injected animals (supplementary material Figs S4B, S3; Movie 2). Regular confocal pictures taken from 34 hpf zebrafish embryos treated with 3 different MDGA2A morpholino oligonucleotides, confirmed that cohesion between trigeminal neurons is weakened in MDGA2A knockdown fish and that some cells undergo ectopic migration (supplementary material Fig. S4A,D). While in confocal stacks of wt and control morpholino injected embryos an average of 12.35±0.21 neurons can be seen in the trigeminal nerve cluster, MDGA2A knockdown fish have a significantly reduced number (9.66±0.27; p<0.005). These missing cells can be found at ectopic positions often along the trigeminal nerve (arrows in supplementary material Fig. S4A), a situation never observed in wt embryos.

**Fig. 5.**
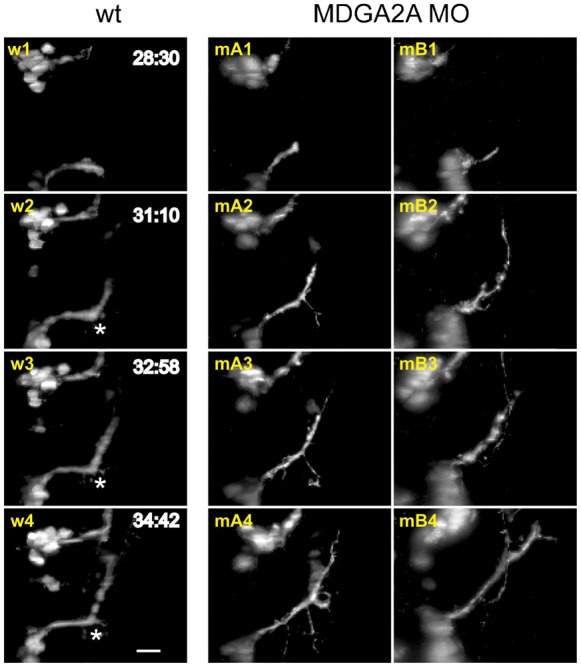
MDGA2A knockdown decreases nVII axon bundling. Representative images from control (w) and MDGA2A morpholino treated (mA and mB) embryos are shown. 28 to 34 h post fertilization facial neurons in wt embryos project axons along a predetermined path, displaying a well-documented 60° turn (asterisks). In MDGA2A morphants this turning angle is absent or strongly reduced and defasciculation events occur much more frequently. Analyzing fluorescence intensity below the main axon bundle of the facial nerve indicates that fluorescence in MDGA2A knockdown embryos is slightly increased, supporting the finding of increased defasciculation and axon branching of facial neurons (see supplementary material Fig. S3D–F). The scale bar represents 20 µm.

Axonal outgrowth of nVII axons started around 28 hpf and 3 h later a characteristic 60° turn could be observed, as previously reported by Higashijima et al. (Higashijima et al., 2000) (Fig. 5, w2–w4, asterisks; supplementary material Movie 1). At the turning point continuous outgrowth of short protrusions were observed, but they readily retracted and the main axon bundle stayed fasciculated (Fig. 5, w2–w4; supplementary material Movie 1). By 34 hpf, a stable bundle of axons all showing the 60° turn was established. However, upon morpholino mediated knockdown of MDGA2A, the turn of the axon was less pronounced and the turning angle was decreased (Fig. 5, mA2-4, mB2-4; supplementary material Movie 3). As for control embryos, axons in the region of the turning point started to protrude in random directions, but in MDGA2A knockdown animals these axons did less frequently retract, and some even continued growing. In addition, the axon bundles in MDGA2A morpholino treated embryos were less organized and often highly defasciculated compared to control embryos (supplementary material Movie 3). To quantify this axon outgrowth phenotype, we measured the fluorescence intensity in an area below the axon in the DSLM images (supplementary material Fig. S3). In animals older than 30 hpf the total fluorescence intensity in this area was slightly higher for MDGA2A morpholino treated embryos compared to control. Confocal pictures of embryos treated with 3 different MDGA2A morpholinos showed extensive protrusions at the choice point and a significantly reduced turning angle (32.75±1.53°) compared to control fish (58.15±1.11, p<0.005; supplementary material Fig. S4B,D).

In summary, in MDGA2A morpholino treated embryos several subpopulations of branchiomotoneurons display errors in the predetermined migration pattern and/or their axons show increased collateral branch formation and less intense bundling.

## DISCUSSION

MDGA proteins have been studied in humans (De Juan et al., 2002; Díaz-López et al., 2005), rats (Litwack et al., 2004), mice (Takeuchi et al., 2007), chickens (Fujimura et al., 2006) and medaka (Sano et al., 2009). Here we identified and cloned three MDGA orthologs in zebrafish, MDGA1, MDGA2A and MDGA2B. We found MDGA2A to be expressed in a subset of motoneurons, especially in the ones of the cranial, trigeminal and facial nerves. Morpholino mediated knockdown of MDGA2A led to aberrant cell migration of trigeminal neurons and to defasciculation and increased branch formation of the trigeminal as well as facial nerve. These results demonstrate that MDGA2A interactions are necessary for proper migration, axon outgrowth and bundling in cranial motoneurons.

In agreement with our current findings, MDGAs in other species have already been implicated in neuronal migration and axon guidance. In rats, MDGA positive cells were found in the pontine migratory stream, suggesting that these circumferentially migrating neurons may rely on this cell adhesion molecule for proper neuronal migration (Litwack et al., 2004). In addition, in MDGA1 loss of function mice proper radial migration of superficial layer cortical neurons is blocked (Takeuchi and O'Leary, 2006), and MDGA-2 knockdown in chicken by RNA interference induced strong axon outgrowth phenotypes in MDGA expressing commissural interneurons (Joset et al., 2011). Instead of turning rostral after crossing the ventral midline, commissural axons in MDGA2A knockdown animals stall at the contralateral side, unable to follow ipsilateral-projecting axon fascicles (Joset et al., 2011).

In zebrafish embryos MDGA2A is expressed in motoneurons of the oculomotor (nIII) and trochlear (nIV) cranial nerve in the midbrain, and the branchiomotoneurons of the trigeminal, facial and vagal nerve in the hindbrain. In support of a role in axon outgrowth, MDGA2 is expressed in the spinal cord during neuronal migration and axon path finding in various species.

Only few MDGA binding partners have been discovered so far. We found earlier that chicken MDGA2 formed homophilic trans-interactions in multiple assays, while heterophilic interactions between MDGA2 and MDGA1 were not detected (Joset et al., 2011). MDGA1 in contrast, did not form homophilic interactions but soluble recombinant protein was shown to bind to axon rich regions in chicken and this interaction was MAM domain dependent (Fujimura et al., 2006; Joset et al., 2011). More recently, it was reported by two independent groups that both MDGAs interact via their Ig-repeats with neuroligin-2 in cis (Lee et al., 2013; Pettem et al., 2013). The affinity of MDGA1 for neuroligin-2 was in the low nanomolar range, while MDGA2 binding was weaker (Pettem et al., 2013). Taken together, these results suggest that MDGA1 and MDGA2 binding preferences clearly differ. MDGA1 may undergo strong heterophilic interactions in cis thereby regulating the function of its binding partners, while MDGA2 may preferentially form homophilic interactions in trans serving as an adhesion factor. Depending on the concentration of MDGA2 in trans and the presence of binding partners such as neuroligin-2 in cis, MDGA2s regulatory or adhesive function may dominate.

Recently, it has been shown that neuron to neuron as well as neuron to extracellular matrix contacts are important for facial motoneuron migration (Wanner et al., 2013). While contact to a specific pioneer neuron seems to be required to lead following facial neurons in the early phase of migration, interaction with axons of the medial longitudinal fascicle is required in a subsequent phase of migration (Wanner and Prince, 2013). In the case of facial branchiomotoneuron migration, cdh2 and Tag1 seem to play a crucial role (Wanner and Prince, 2013; Sittaramane et al., 2009); however, these molecules have no influence on trigeminal neuronal migration. In our case the knockdown of MDGA2A has no effect on neuron migration but enhances the mobility of trigeminal neurons, which relocated from their original place to settle at ectopic positions. This increased mobility might be due to the lack of MDGA2A mediated homophilic interactions in trans, weakening the cohesion of trigeminal neurons enabling them to migrate along their axonal fascicle. Interestingly, even though facial neuronal migration is unaffected in MDGA2A knockdown animals, axonal outgrowth of facial neurons is clearly impaired. In line with the fact that MDGA-2 is a homophilic cell adhesion molecule, the MDGA2A positive facial nerve is less compact and seems to contain fewer axons in MDGA2A morphants. In addition, increased axon defasciculation along the facial nerve can be seen and the entire nerve path deviates from patterns seen in wild type embryos, again suggesting that MDGA2A mediated homophilic adhesion is keeping the facial nerve compact. Moreover, at a well-defined choice point, where trigeminal axons in wild type animals make a characteristic 60° turn (Higashijima et al., 2000), axons in MDGA2A knockdown animals display many collaterals and large protrusions, as being unable to make the correct pathway decision. Consequently, the angle at which axons in MDGA2A knockdown animals are leaving this choice point is dramatically reduced, suggesting that some aspects of proper guidance are missing. A similar phenotype has already been observed in MDGA2 knockdown chicken embryos, where turning of commissural interneurons after midline crossing is impaired. Instead of turning rostral, commissural axons in MDGA2A deficient embryos stall after midline crossing being unable to interconnect with MDGA2A positive tracts on the contralateral site (Joset et al., 2011). This similarity between the chicken and our zebrafish phenotype suggests that MDGA2A might confer adhesive interactions between different axonal tracts, thereby enabling follower tracts to use pioneer tracts as predetermined highways.

Interestingly, rare deletions in the MDGA2 gene were recently correlated with autism spectrum disorders (ASD) (Bucan et al., 2009). This puts MDGA2 in line with other neuronal cell adhesion molecules of the immunoglobulin family, such as contactins, NRCAM, CADM1 and LRFN5 that are implicated in axon migration and guidance and were associated with autism (Berglund et al., 1999; Fernandez et al., 2004; Glessner et al., 2009; Roohi et al., 2009; Cottrell et al., 2011; Morrow et al., 2008; van Daalen et al., 2011; Bonora et al., 2005; Marui et al., 2009; Zhiling et al., 2008; de Bruijn et al., 2010). In summary, the association of truncated MDGA2 variants with ASD, and the notion that a number of neuronal cell adhesion factors are implicated in ASD, supports also a role of human MDGA2 as a cell adhesion molecule important in neuronal positioning and axon guidance.

## MATERIALS AND METHODS

### Fish maintenance and breeding

Wild-type fish from the inbred WIK and Tübingen strain as well as fish from the Islet1-GFP transgenic line were bred and maintained under standard conditions. Embryos were raised at 28°C in E3 medium. Morphological features characteristic for developmental stages were used to determine the stages of the embryos in hours post fertilization (hpf), according to Kimmel et al. (Kimmel et al., 1995). All experiments were performed according to the European Communities Council Directive for animal use in science (2010/63/EU) and in accordance with Swiss laws.

### Identification and cloning of zebrafish MDGAs

Through comparative database searches with the corresponding rat and chicken MDGA sequences we identified and subsequently cloned cDNA sequences of three zebrafish MDGA orthologs, MDGA1, MDGA2A and MDGA2B, respectively. Oligo dT primed cDNA, serving as template for MDGA PCRs, was done using the first strand cDNA kit (Invitrogen, Carlsbad, CA). Total RNA used for reverse transcription was isolated from 5 day old wt fish using the QIAShredder and the RNeasy kit (Qiagen, Hombrechtikon, Switzerland). For polymerase chain reaction (PCR) Taq polymerase (Taq Gold; Applied Biosystems) and sequence-specific oligonucleotide primers were used. Amplified DNA pieces were subcloned into the TOPO pCRII vector (TA Cloning Kit Dual Promoter, Invitrogen, Carlsbad, CA) and subsequently sequenced.

### Whole mount in situ hybridization

Linearized MDGA containing plasmids were purified using the QIAquick PCR Purification Kit (Qiagen). *In vitro* transcription of DNA probes using the SP6 and T7 RNA polymerases was performed using the Roche DIG-RNA Labeling Kit (Roche Diagnostics, Rotkreuz, Switzerland). Probes longer than 1000 bp were hydrolyzed prior to hybridization. Embryos predetermined for in situ hybridization were treated with 3 µM PTU [1- phenyl-2-hiourea (Sigma)] to suppress pigmentation. PTU-treated embryos were collected at different stages of development and staged by morphology. The embryos were dechorionated, fixed in paraformaldehyde (4% in PBS, pH 7.25) and incubated at 4°C overnight or at room temperature (RT) for 1–2 h. Subsequently, the embryos were dehydrated in a PBT/methanol series and stored in methanol at −20°C. For in situ hybridization, embryos were rehydrated stepwise in methanol/PBT. To enhance penetration of the antisense RNA probe, embryos older than 24 hpf were treated with 10 µl/ml Proteinase K (Roche) at different durations, depending on their developmental stage. The reaction was stopped by rinsing in PBT (PBS pH 7.25, 0.1% Tween-20). Then, the embryos were post-fixed in paraformaldehyde (4% in PBS, pH 7.25) for 20 min and washed in PBT 5 times for 5 min. Embryos were prehybridized for 2–5 h between 62 to 68°C in hybridization buffer (50% formamide, 5×SSC, 5 mg/ml torula yeast RNA (typeVI, Sigma), 50 µg/ml heparin (Sigma), 125 µg/ml fish sperm DNA (Roche), 0.1% Tween-20). Afterwards, the prehybridization buffer was replaced by pre-warmed hybridization buffer containing 1–2 ng/µl of Dig labeled antisense RNA. Hybridization occurred at 62°C–68°C overnight. The embryos were washed in a series of hybridization buffer/SSC steps for 15 min at 62 to 68°C. Embryos were subsequently washed in MABT (100 mM Maleic acid, 150 mM NaCl, 0.1% Tween-20, pH 7.5) for 5 min at RT and blocked for 2 h in blocking solution (2% Boehringer Blocking reagent in MABT). Antibody solution (anti-Digoxigenin-AP, Fab fragments from Roche diluted 1:4000 in blocking solution) was incubated ON at 4°C, followed by 3×15 min washes in blocking solution and 3×15 min in NTMT (0.1 M Tris-HCl pH 9.5, 0.1 M NaCl, 0.05 M MgCl, 1 mM Levamisol, 0.1% Tween-20). Staining solution (0.5 mg/ml NBT, 0.175 mg/ml BCIP (both from Roche) in NTMT) was applied for 1–4 h in darkness, and was replaced by PBT. Embryos were post-fixed in 4% PFA for 20 min, washed in PBT and brought into Glycerol for imaging and storage. For obtaining optimal pictures, larvae were mounted on an adapted glass slide in 100% glycerol (Sigma-Aldrich) and the DIC modus of a light microscope (Olympus BX61) and a colour camera (ColorView IIIu, Soft Imaging System, Olympus) were used.

### Immunohistochemistry

Embryos were fixed in 4% PFA for 2 h at RT and brought into Methanol at −20°C via dilution series, for at least 1 h or for long term storage. Embryos were rehydrated in a Methanol series and washed 5× in PBT (PBS pH 7.25, 0.1% TritonX-100) before permeabilization in Collagenase A solution (3 mg/ml in Ringer solution) for 150 min at RT. Embryos were than washed twice in PBT, fixed in 4% PFA for 20 min, washed 5× in PBT and blocked at RT for several hours in blocking solution (1% BSA, 1% DMSO, 0.5% TritonX-100, 2.5% Normal goat serum, in PBS). Antibodies (rabbit anti-MDGA2A 1:300, mouse anti-GFP (Roche) 1:200 in blocking solution) were applied and incubated ON at 4°C. Embryos were then washed 4×15 min in PBT, 3×15 min in blocking solution and incubated in secondary antibody solution (Alexa goat anti-rabbit 568 1:500, Alexa goat anti-mouse 488 1:1000 in blocking solution) for 1 h at RT. After 4×15 min washes in PBT, embryos were brought into Glycerol for imaging and storage (at 4°C). To demonstrate the effectiveness of MDGA2A morpholino protein knockdown, wt and morphants were stained and imaged under identical conditions (e.g identical exposure times).

### Antisense morpholino oligonucleotide injections

Antisense morpholino oligonucleotides were synthesized by Gene Tools (Gene Tools, LLC, Philomath, OR). The targeting sequence for MDGA2A-MO-A was 5′- ACAGGAGAGCCCAGATAATATCCAT-3′ covering the first 25 nucleotides of the coding sequence (1–25). Targeting sequences of MO-B and MO-C were 5′-CGTTAGTCTGCACATATCCAGCTCC-3′ (−31 to −6) and 5′-TCTTCACGTTAGTCTGCACATATCC-3′ (−24 to 1) respectively. Standard control morpholinos 5′- CCTCTTACCTCAGTTACAATTTATA-3′ were used as control to monitor unspecific developmental defects. 4.5 ng (MO-A) to 6 ng (MO-B,C, control) of antisense morpholino oligonucleotides were injected into one- to four-cell stage of Islet1-GFP embryos. After injections, 250 µl Penicillin per dish was added. Dechorionated fish were fixed in paraformaldehyde (4% in PBS, pH 7.25) at different stages followed by dehydration in a PBT/methanol series and stored in methanol at −20°C. The fish were analyzed by immunohistochemistry and light sheet microscopy.

### Production of anti-MDGA2A peptide antibodies

For the generation of MDGA2A specific antibodies, a short peptide covering the amino acids 276–289 (LSWVRNTEELPKKS) of MDGA2A was synthesized (Eurogentec, Belgium). The used sequences were checked for intrafamiliar sequence homology using the MegAlign program. Prior to immunization, pre-sera were tested for cross-reactivity by western blot analysis. Two rabbits were injected with the synthesized peptide. Rabbits were boosted after 14 days and 28 days, respectively. A first blood sample was taken at day 38, followed by an additional injection on day 56 and a second blood sample that was taken at day 66. The final bleeding was done after 87 days. Antibodies were delivered as sera and IgGs affinity purified against the corresponding MDGA2A peptide.

### Confocal analysis

Fluorescent samples were mounted in GG1 Glycerol Gelatin (Sigma) and viewed with a Leica SP2/SP8 Confocal Microscope. 20× and 63× Glycerin objectives were used to picture confocal sections every approximate 0.6 µm with an average number of 150 steps.

### Light sheet microscopy

We employed Digital Scanned Laser Light Sheet Microscopy (DSLM) for three-dimensional imaging, as previously described (Keller et al., 2008). The central thickness of the light sheet was set to 4 µm (FWHM). An Achroplan W 40×/0.8 water-dipping objective (Carl Zeiss) was used for fluorescence detection. Three-dimensional image stacks were recorded in 16 min time intervals with a Coolsnap 4K CCD camera (12 bit, 7.4 µm pixel pitch, 2048 × 2048 pixels; Roper Scientific). The spacing between single images in the three-dimensional stack was set to 2.2 µm and a total of 600 images were recorded per stack, covering a specimen volume of 379 × 379 × 1320 µm.

### Image processing

Images were deconvolved with the Lucy-Richardson algorithm and specimen drift was corrected by custom software developed in Matlab, as previously described (Keller et al., 2008). For visualization of the three-dimensional image data as a function of time, maximum-intensity projections were generated.

### Intensity measurement

To measure the intensity of axons, a line was drawn manually along the axon, a region in a certain radius around that line was selected and the pixel intensities of that region in the maximum projection were averaged. An area at the edge of the image was used for background subtraction. This procedure was repeated for every maximum projection of a movie to obtain the time course of fluorescence intensity. The values from multiple embryos were averaged and plotted together with the standard error of the mean.

### Statistical analysis

Significance of fluorescent intensity along the facial nerve was calculated by bootstrapping. For each time point the mean and confidence intervals at an alpha value at 0.05 were calculated from 1000 repeats. For determining the number of cells in nV clusters wt (n = 11), control morpholino injected (n = 6) and MDGA2A morphant (n = 28) zebrafish in 3D reconstructions of confocal stacks using Bitplane Imaris software were analyzed. Statistical analysis was performed using two-tailed homoscedastic Student's t-test. For the analysis of migrating cells a two-tailed heteroscedastic Student's t-test was done because no migrating cells have been observed in wt zebrafish. The angle of nVII projections (wt n = 9, control morpholino injected n = 6, and MDGA2A morphants n = 26) were measured using Image J's angle tool on maximum intensity projections. Statistical significance was calculated by two-tailed homoscedastic Student's t-test.

## Supplementary Material

Supplementary Material
