# Supplementary material for: Proper migration and axon outgrowth of zebrafish cranial motoneuron subpopulations require the cell adhesion molecule MDGA2A
Source: Biol Open. 2015 Jan 8;4(2):146–54. doi: 10.1242/bio.20148482 (PMC4365483; doi:10.1242/bio.20148482)
Supplement: Supplementary Material [file supp_4_2_146__index.html]

Proper migration and axon outgrowth of zebrafish cranial motoneuron subpopulations require the cell adhesion molecule MDGA2A — Supplementary Material 

# Proper migration and axon outgrowth of zebrafish cranial motoneuron subpopulations require the cell adhesion molecule MDGA2A

## bio.20148482 Supplementary Material

**Files in this Data Supplement:**

- Supplementary Material - Esther Ingold et al. doi: 10.1242/bio.20148482
- Movie 1 - **Movie 1. In vivo development of cranial motoneurons observed by light sheet microscopy.** Zebrafish islet-GFP wt larva were monitored between 24−36 hpf. Fluorescent images were taken every 16 min and processed as described in material and methods. Scale bare equals 20 µm.
- Movie 2 - **Movie 2. Development and migration of trigeminal motoneurons in wild type and MDGA2A knockdown animals observed by light sheet microscopy.** The left panel shows the development of trigeminal neurons during the time between 24 and 36 hpf in islet-GFP fish. Note that neurons within the trigeminal cell cluster remain tightly together, sending out axons into the trigeminal nerve. The right panel depicts the situation in MDGA2A knockdown animals. In the case of MDGA2A knockdown increased mobility and intense migration of trigeminal neurons along the trigeminal nerve can be observed. Scale bar represents 20 µm.
- Movie 3 - **Movie 3. Development of the facial nerve in wild type and MDGA2A knockdown animals.** Wild type and MDGA2A knockdown islet-GFP fish were monitored by light sheet microscopy between 24 and 36 hpf. The left panel illustrates the normal development of the facial nerve during this period of development. Around 30 hpf the facial nerve in wt larva displays a characteristic 60˚ turn. At this ‘‘choice point’’ temporary stalling and increased transient branching can be observed even in the wild type. However, most branches retract over time and the axon bundle stays fasciculated. The middle and the right panel depict facial nerve growth in MDGA2A knockdown animals. Note that the typically observed 60˚ turn is absent in MDGA2A morphants and that strongly increased branching and defasciculation along the facial nerve is seen in these larva. Scale bar equals 20 µm.
