# Supplementary material for: Proper migration and axon outgrowth of zebrafish cranial motoneuron subpopulations require the cell adhesion molecule MDGA2A
Source: Biol Open. 2015 Jan 8;4(2):146–54. doi: 10.1242/bio.20148482 (PMC4365483; doi:10.1242/bio.20148482)
Supplement: Supplementary Material [file supp_bio.20148482_bio.20148482-s1.pdf]

Biology Open (2015) 000, 1–9 doi:10.1242/bio.20148482

Supplementary Material

Esther Ingold et al. doi: 10.1242/bio.20148482

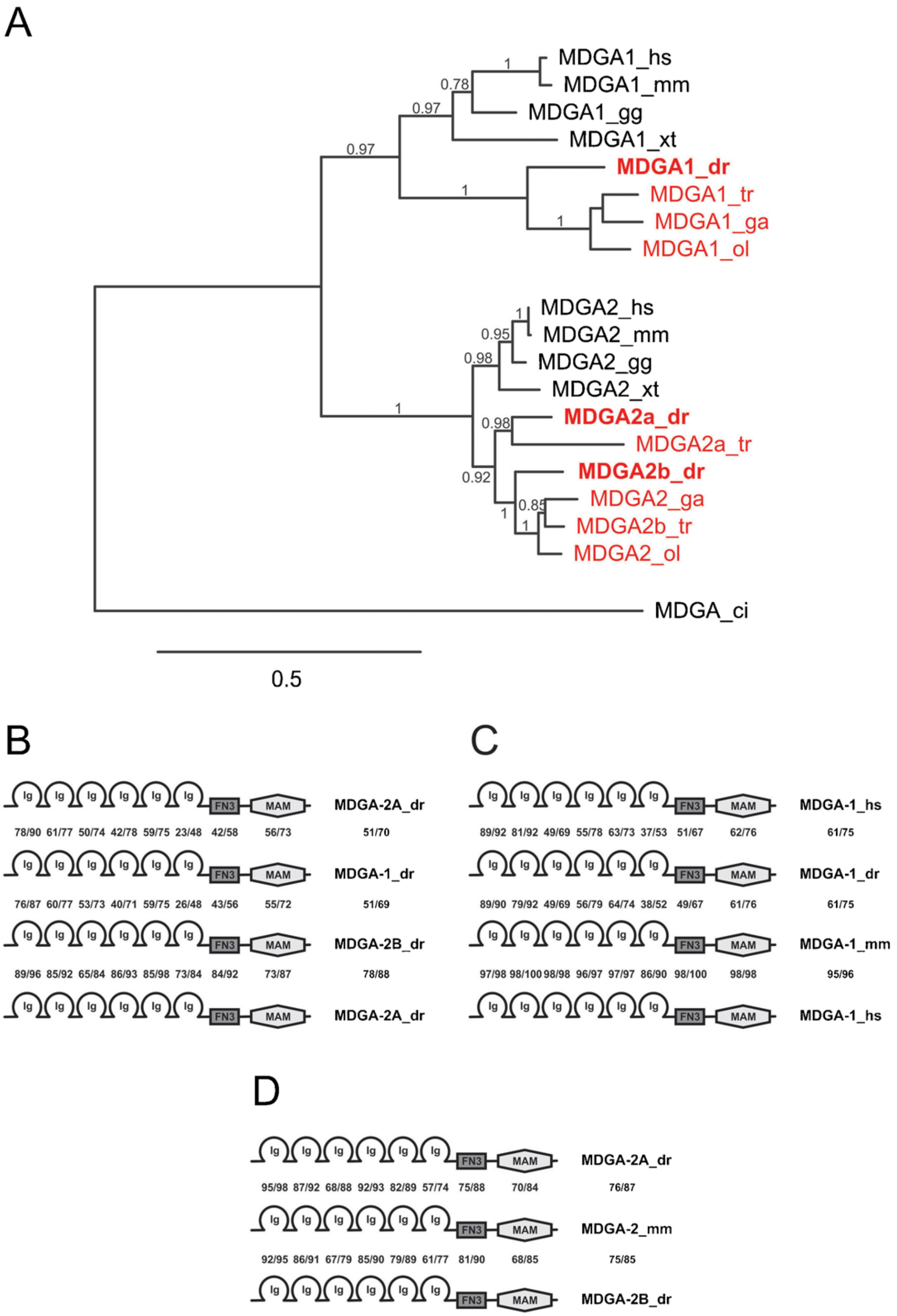

Fig. S1. Phylogenetic relationship and homology between MDGA proteins. (A) Maximum likelihood phylogeny of members of the zebrafish (dr), fugu (tr), medaka (ol), stickleback (ga), mouse (mm), Xenopus (xt), chicken (gg) and human (hs) MDGA family. The phylogenetic tree was build using 722 representative amino acids determined by the program Gblocks after sequence alignment using MUSCLE. Bootstrap values above 50% (0.5) are shown. Fish MDGA proteins are shown in red and zebrafish proteins are highlighted in bold. As an outgroup to root the tree ciona (ci) MDGA was used. Note that in zebrafish and fugu MDGA-2 genes have retained their duplicates after the teleost specific whole genome duplication, resulting in an A and B paralog. The scale bar shows the percentage (0.5 equals 50%) of amino acid substitutions required to generate the corresponding tree. (B) Homology comparison between individual domains of zebrafish MDGAs. Domain boundaries were identified using the smart program (http://smart.embl-heidelberg.de/) and subsequently compared pairwise using BLASTP. While the first number represents the percentage of identical residues, the second number indicates the number of conserved residues (eg. L vs. V; D vs. E; etc.). Note that the amount of conservation varies greatly between the different domains. (C) Conservation between human, mouse and zebrafish MDGA1s. (D) Conservation between mouse and zebrafish MDGA2s. Note that comparison between human and zebrafish MDGA2s gave similar results.

Biology Open (2015) 000, 1–9 doi:10.1242/bio.20148482

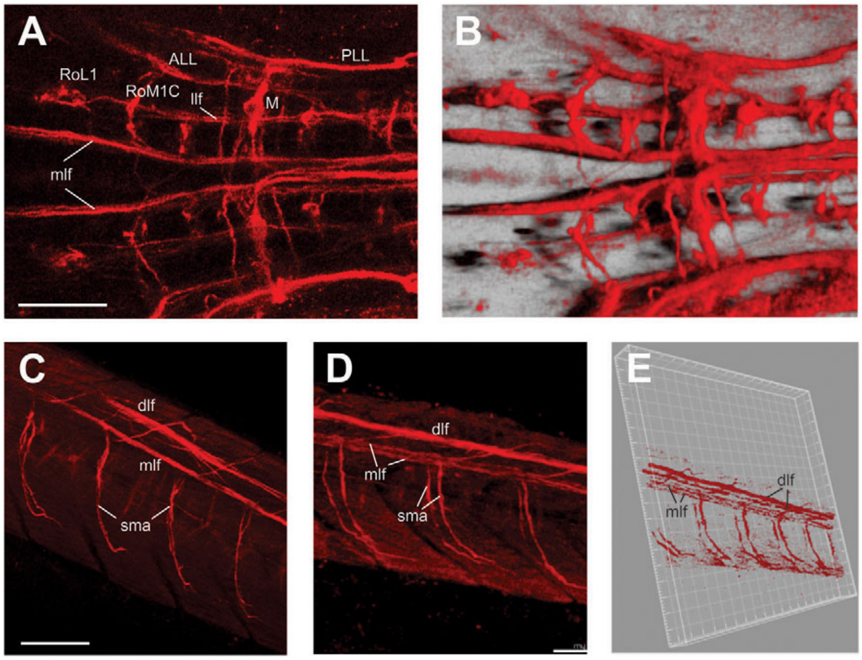

Fig. S2. MDGA-2A antibodies stain multiple tracts in the zebrafish hindbrain and spinal cord. (A) A ladder-like staining of MDGA-2A positive reticulospinal interneurons can be observed. Their MDGA-2A labeled axons project into one of the two major longitudinal fiber pathways through the CNS, the medial longitudinal fascicle (mlf) or the lateral longitudinal fascicle (llf). In the hindbrain, the mlf splits into dorsal and ventral components (mlfD and mlfV, respectively). (B) 3D reconstruction of the area above generated with the Imaris software. (C,D) MDGA-2A positive longitudinal tracts (dlf and mlf) extend into the spinal cord. At each somite segment, the MDGA-2A antibody stains spinal motor axons (sma) emerging from the mlf. (E) A 3D reconstruction of the are depicted in C,D generated with the Imaris software. Scale bars equal 200 µm.

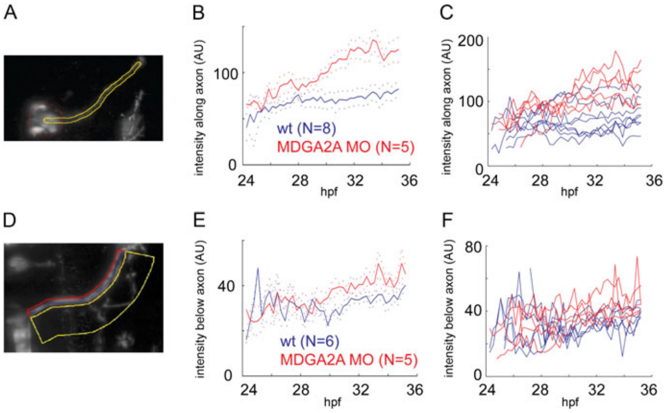

Fig. S3. Detailed quantification of the MDGA-2A phenotypes.
(A) Fluorescence intensity in the region along the axon (yellow area) was measured in wt and MDGA-2A knockdown animals. Background fluorescence intensity in an area of identical size but outside the migration path of axons and neurons was subtracted to obtain the net intensity in the area of interest. (B) The fluorescence intensity along the trigeminal axon bundle was quantified in 8 control embryos and 5 MDGA-2A morpholino treated embryos. Shown are the mean and error of the mean (as dotted lines). Due to aberrant neuronal migration along the trigeminal axon bundle staining intensity in this area increases clearly in MDGA-2A knockdown animals. (C) Individual measurements of wt and MDGA-2A knockdown are shown. (D) Fluorescence intensity in an area below the axon bundle of the facial nerve is increased in MDGA-2A knockdown animals. Fluorescence intensity in a region underneath the facial axon bundle (yellow area) was measured in wt and MDGA-2A knockdown animals. Background fluorescence intensity in an area of identical size but outside the projection path of axons was subtracted to obtain the net intensity in the area of interest. (E) In MDGA-2A knockdown animals older than 30 hpf, the mean fluorescence intensity underneath the facial nerve was increased, representing increased branching and defasciculation. Note that in wt animals residual fluorescence intensity can also be detected, as transient branch formations also occur during regular development. (F) Individual measurements of wt and MDGA-2A knockdown are shown.

Biology Open (2015) 000, 1–9 doi:10.1242/bio.20148482

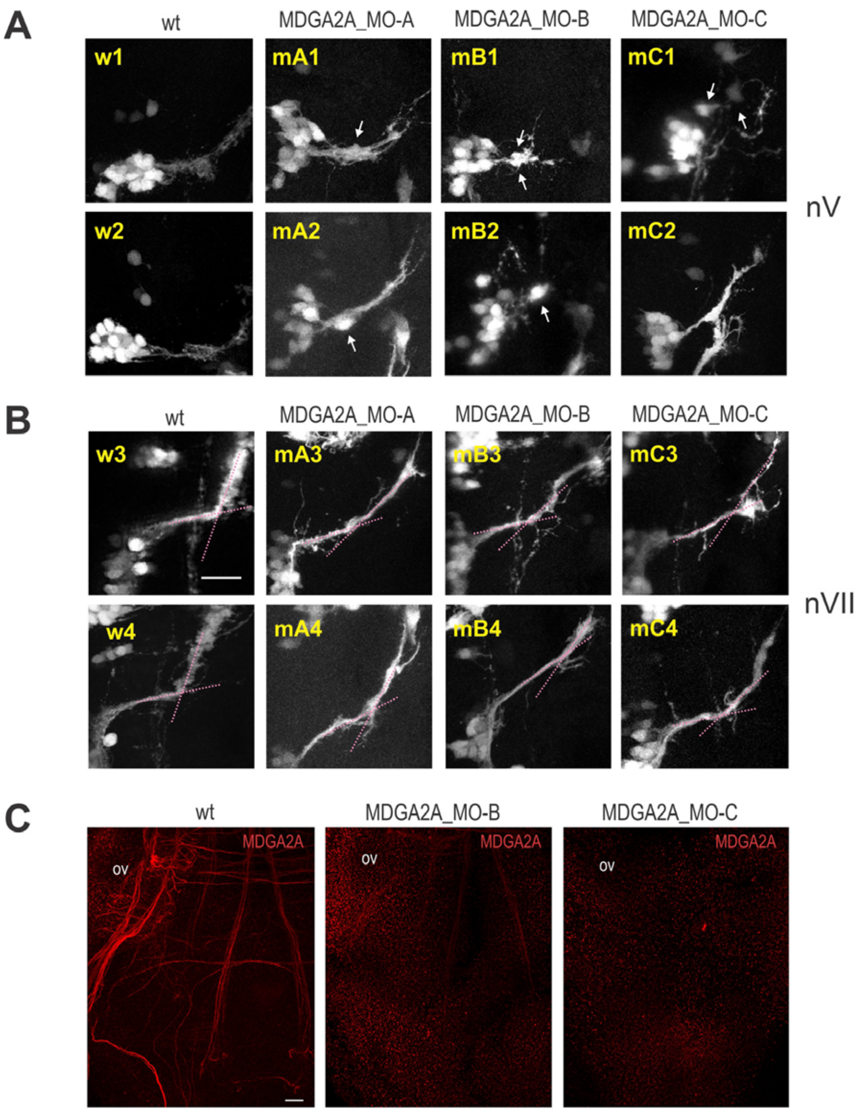

D

	n nV clusters	Average cells in nV	Average cells along projcetion	n turning angles	Average turning angle
Uninjected Isl1_GFP	11	12.18±0.30	0	9	57.14±1.38°
Standard Control MO	6	12.66±0.21	0	6	59.67±1.82°
MDGA2A_MO-A	10	9.20±0.61	2.50±0.20	9	32.63±1.78°
MDGA2A_MO-B	8	9.38±0.53	1.88±0.61	9	28.36±1.88°
MDGA2A_MO-C	10	9.41±0.40	0.90±0.28	8	37.83±3.44°
**Sum Control**	17	12.35±0.21	0	15	58.15±1.11°
**Sum MDGA2A_MOs**	28	9.35±0.32	1.75±0.38	26	32.75±1.53°

Fig. S4. Different MDGA2A morpholino oligonucleotides located around the ATG start codon cause identical phenotypes. (A) Morpholino induced MDGA2A knockdown increases mobility of trigeminal neurons. Representative images from wild type and zebrafish embryos treated with different MDGA2A morpholinos are shown. At 34 hpf trigeminal neurons in wild type embryos have formed a dense trigeminal cell cluster and have send out axons towards their targets (w1,w2). In animals treated with different MDGA2A start-site morpholinos the compactness of the trigeminal cell cluster is impaired (mA1/2, mB1/2, mC1/2). Trigeminal neurons migrate along their axon bundles leaving their place of origin (arrows). (B) MDGA2A knockdown influences axon turning and bundling of the facial nerve. Representative images from control (w3, w4) and MDGA2A morpholino treated embryos (mA3/4, mB3/4, and mC3/4) are shown. 34 h post fertilization facial neurons in wt embryos project axons along a predetermined path, displaying a 60˚ turn (pink dotted lines) at a well-documented turning point. In the different MDGA2A morphants this turning angle is strongly reduced and the formation of axon collaterals occurs much more frequently around this turning point. The scale bar represents 20 µm. (C) MDGA2A morpholinos efficiently reduce MDGA2A protein expression. wt and MDGA2A morpholinos treated embryos were stained with MDGA2A antibodies to visualize axonal tracts. In wt embryos tracts are clearly stained. Under identical staining and recording conditions axonal staining in MDGA2A knockdown animals is absent or very weak, demonstrating that the MDGA2A protein in these animals is strongly downregulated. As a landmark the otic vesicle (ov) is highlighted. The sequence of the morpholinos used (MDGA2A_MO-B/C) is given in the material and methods section. The scale bar represents 20 µm. (D) Statistical analysis of migration and axon guidance defects in MDGA2A knockdown animals. The number of analyzed trigeminal cell clusters and facial nerves for uninjected wild type, standard control and MDGA2A (MO-A, MO-B, MO-C) morpholino injected animals is given. The average cell number in the trigeminal cell cluster as well as the average turning angle of the facial nerve under the different experimental conditions are summarized. Note that the last two lines represent the pooled data of control (wt and standard control morpholino injected embryos) and MDGA2A (MO-A, MO-B, MO-C) morpholino injected animals.

Biology Open (2015) 000, 1–9 doi:10.1242/bio.20148482

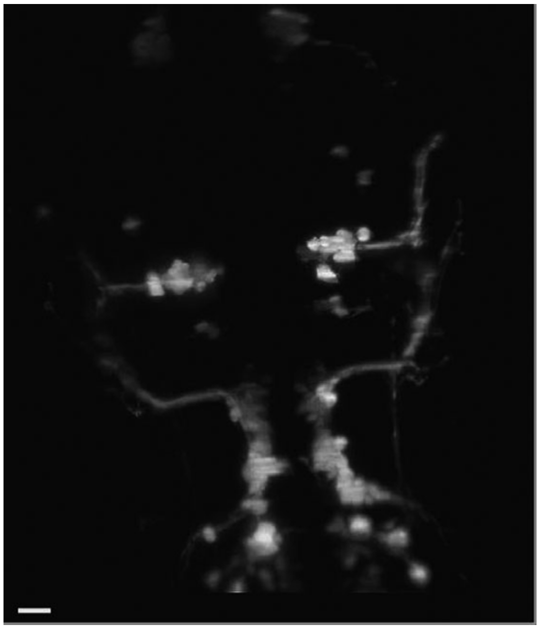

Movie 1. In vivo development of cranial motoneurons observed by light sheet microscopy. Zebrafish islet-GFP wt larva were monitored between 24–36 hpf. Fluorescent images were taken every 16 min and processed as described in material and methods. Scale bare equals 20 µm.

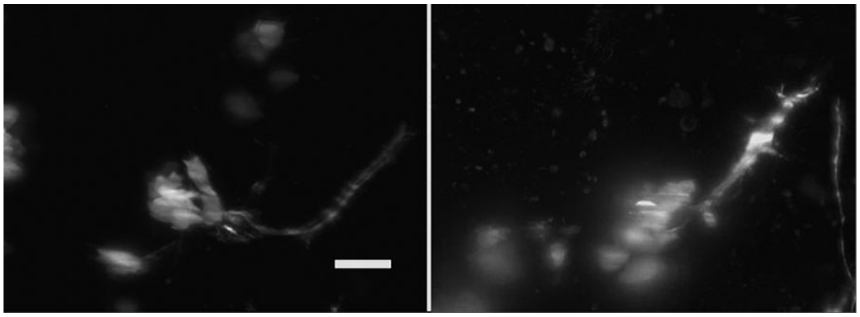

Movie 2. Development and migration of trigeminal motoneurons in wild type and MDGA2A knockdown animals observed by light sheet microscopy. The left panel shows the development of trigeminal neurons during the time between 24 and 36 hpf in islet-GFP fish. Note that neurons within the trigeminal cell cluster remain tightly together, sending out axons into the trigeminal nerve. The right panel depicts the situation in MDGA2A knockdown animals. In the case of MDGA2A knockdown increased mobility and intense migration of trigeminal neurons along the trigeminal nerve can be observed. Scale bar represents 20 µm.

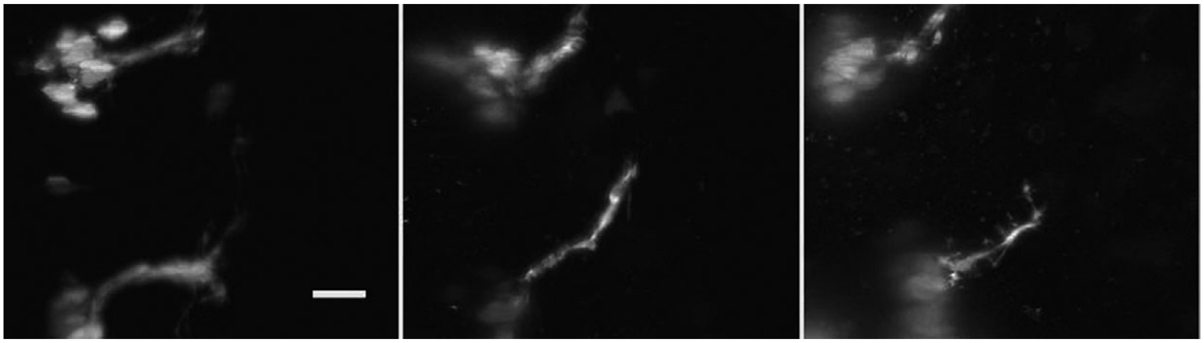

Movie 3. Development of the facial nerve in wild type and MDGA2A knockdown animals. Wild type and MDGA2A knockdown islet-GFP fish were monitored by light sheet microscopy between 24 and 36 hpf. The left panel illustrates the normal development of the facial nerve during this period of development. Around 30 hpf the facial nerve in wt larva displays a characteristic 60˚ turn. At this ''choice point'' temporary stalling and increased transient branching can be observed even in the wild type. However, most branches retract over time and the axon bundle stays fasciculated. The middle and the right panel depict facial nerve growth in MDGA2A knockdown animals. Note that the typically observed 60˚ turn is absent in MDGA2A morphants and that strongly increased branching and defasciculation along the facial nerve is seen in these larva. Scale bar equals 20 µm.
